# Case Report: Rapid cataract development preceding diabetes mellitus in *WFS1* spectrum disorder

**DOI:** 10.3389/fopht.2025.1612964

**Published:** 2025-09-09

**Authors:** Aaishwariya A. Gulani, Courtney Duggan, Danielle M. Ledoux, Eric D. Gaier

**Affiliations:** 1 Hamilton Eye Institute University of Tennessee, Memphis, TN, United States; 2 Massachusetts Eye and Ear, Boston, MA, United States; 3 Specialized Pediatric Eye Care, Beverly, MA, United States; 4 Department of Ophthalmology, Boston Children’s Hospital, Boston, MA, United States; 5 Department of Ophthalmology, Harvard Medical School, Boston, MA, United States; 6 Picower Institute for Learning and Memory, Massachusetts Institute of Technology, Cambridge, MA, United States

**Keywords:** WFS1 spectrum disorder, Wolfram syndrome, cataract, diabetes mellitus, optic atrophy

## Abstract

*WFS1* spectrum disorder is a rare condition, characterized by diabetes insipidus, diabetes mellitus, optic atrophy, and deafness (DIDMOAD). A 2-year-old female patient with a history of sensorineural hearing loss presented with rapid, sequential cataract development. Diabetes mellitus was not manifested at the time but developed 4 years later. While cataracts have been described in this syndrome, rapid acquisition of cataracts in the setting of mild hyperglycemia was unique considering they could not be definitively attributed to diabetes mellitus alone. This case provides real-world evidence that rapid *WFS1*-related cataract development may result from the underlying condition in conjunction with or independent from *WFS1*-associated diabetes mellitus.

*WFS1* spectrum disorder (*WFS1*-SD) is a rare genetic condition, characterized by diabetes insipidus, diabetes mellitus, optic atrophy, and deafness (DIDMOAD) (1). Patients typically present with insulin-dependent diabetes mellitus at a median age of 6 years and optic atrophy at 11 years (1). Other manifestations include neurological deficits, primarily ataxia, anterior pituitary dysfunction, and urological complications such as hydroureteronephrosis; most of these present later in life and, thus, are screened for once the genetic diagnosis is made (2). Aside from optic atrophy, ophthalmic manifestations include cataracts, pigmentary retinopathy, and diabetic retinopathy (2). This case provides evidence that rapid *WFS1*-related cataract development may result from the underlying condition perhaps in conjunction with *WFS1*-associated diabetes mellitus.

A 2-year-old female patient was transferred from an outside emergency department because of right eye leukocoria and exotropia. Two weeks prior to presentation, the patient’s mother noticed the patient closing the right eye, which was drifting out more with a “cloudy haze” over the pupil. Examination on presentation revealed visual acuities of light perception OD and 20/160 (Teller) OS. She manifested a constant exotropia and left eye fixation preference (**Figure 1A**). A complete cataract precluded any view to the right fundus. B-scan ultrasound was without retinal detachment. There was optic disc pallor on the left with no significant cataract precluding an adequate view to the fundus.

**Figure 1:**
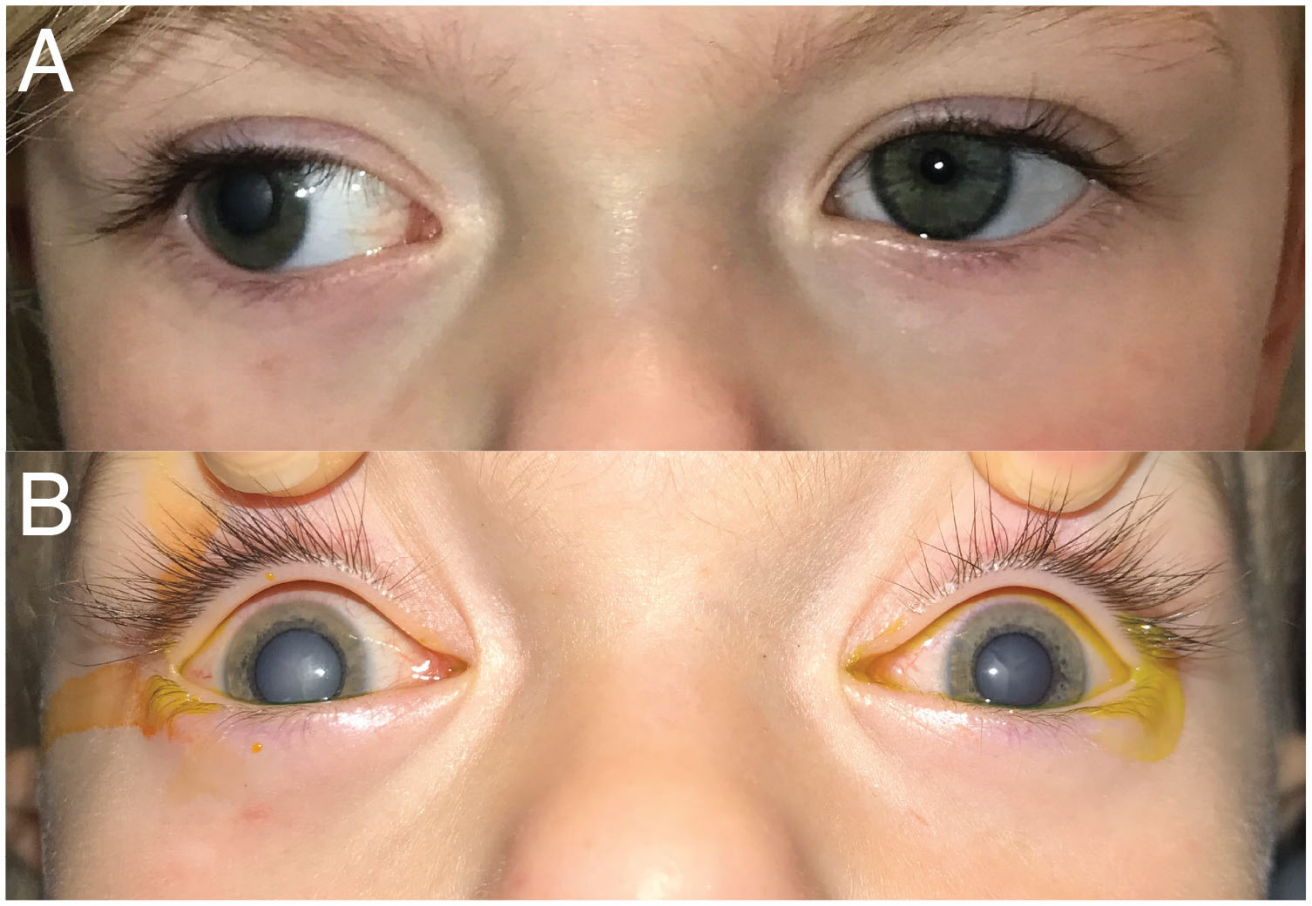
External photographs of exotropia and cataracts. **(A)** Day 0—first exam/presentation. **(B)** Day 5—under anesthesia just prior to cataract extraction on the right.

The patient had been born at term via c-section with APGAR scores of 8 and 9. She had a failed auditory screening at birth that prompted further workup. Infectious screening, including cytomegalovirus and rubella, was negative. Magnetic resonance imaging (MRI) pursued as a part of this workup showed bilateral hypoplastic auditory nerves and no comment on the optic nerves. In addition to congenital hearing loss, she had a malpositioned anus, poor weight gain, and short stature and was referred to genetics for further workup. Workup showed high concentrations of amino acids and hyperammonemia, three auricular tags, and normal eyes with reactive pupils. At age 1, an ophthalmologist diagnosed intermittent exotropia; no other ocular abnormalities were reported. She did not have a relative afferent pupillary defect. Family history was significant for insulin-dependent diabetes mellitus in her mother, uncle, and maternal grandfather. Genetic testing of the patient revealed a heterozygous missense variant of likely risk [NM_006005.3(WFS1):c.2430C>G (p.Phe810Leu); rs1553879021] in *WFS1* (Otogenome, Laboratory for Molecular Medicine). This variant is listed in ClinVar (3) and absent from gnomAD (4), but a variant with the same translational consequence was recently reported as likely pathogenic (5). Multiple *in silico* analyses of the variant were suggestive of pathogenicity. The patient’s mother did not carry the variant, and the father, whose family history is unknown, was not available for testing.

The decision was made to remove the cataract. The right eye cataract extraction with intraocular lens (IOL) placement occurred 5 days after initial presentation to the emergency department. Dilated fundus examination under anesthesia after cataract removal revealed a pale right optic disc. During this exam, it was apparent that the left lens had become involved with a smaller, central cataract (**Figure 1B**). She underwent left cataract extraction with IOL placement 5 weeks later. There was no posterior bulge to either lens to suggest persistent fetal vasculature or posterior lentiglobus. 

One-month post-operative assessment confirmed bilateral pallor with a cup-to-disc ratio of 0.8 OD and 0.4 OS. Visual acuities had improved to 20/130 OD and OS, and she was prescribed bifocal glasses to optimize her visual development. Hemoglobin A1c was 6.1%, 5.9%, and 6.5% at 1, 3, and 4 months post-operatively, respectively, with home fasting finger-stick glucose values of 78–90 mg/dL without treatment. Serum osmolality at that time was normal (286 mOsm/kg). Six months post-operatively, the patient began experiencing polyuria and polydipsia and her hemoglobin A1c was found to be 7.2%. She was referred to a local hospital for admission and initiation of insulin therapy. Three years later, visual acuity with correction was 20/41 with both eyes viewing (Teller; patient would not tolerate monocular occlusion).

Early life cataracts have primarily been associated with dominant *WFS1*-SD (1). Berry et al. isolated a missense mutation in *WFS1* (distinct from our patient’s; c.1385A-to-G in exon 8, E462G) that causes congenital nuclear cataracts (6). De Franco et al. described spontaneous heterozygous *WFS1* variants associated with neonatal diabetes, deafness, and congenital cataracts within the first year of life, a more severe phenotype than typical of *WFS1*-SD (7). Our patient already carried a diagnosis of *WFS1*-SD after genetic testing triggered by identification of sensorineural hearing loss and reportedly had a complete screening eye examination without cataracts. She subsequently developed rapid-onset, sequential cataracts in the setting of prediabetic glycemic screening. Similarly, a recent report describes a Pakistani child who acquired cataracts at age 3, subsequently developed systemic manifestations of diabetes mellitus 2 years later, and harbored a different, novel, heterozygous *WFS1* variant (8). While we cannot exclude the fact that our patient had subtle congenital cataracts that progressed, her normal dilated ophthalmic examination prior to presentation suggests they were acquired.

Outside of *WFS1*-SD, rapid cataract development can be seen in isolated diabetes mellitus, albeit in <1% of cases. However, among pediatric patients who develop diabetes-related cataracts, hyperglycemic severity ranges well beyond those seen in this patient with HbA1c percentages of 9.0% (8.55%–9.38%, *p* < 0.001) in patients with early cataracts (9). The wolframin protein is expressed in the developing lens (6), and it plays various roles in the regulation of endoplasmic reticulum stress and calcium homeostasis (10). These same cellular functions are implicated in the cataract development in diabetes mellitus (11, 12). Taken together, we posit that increased baseline endoplasmic reticulum stress secondary to compromised wolframin function may impart increased susceptibility to hyperglycemic stress in lens cells to yield early and rapid cataract development in the setting of relatively mild glycemic dysregulation. Runaway, vicious-cycle mechanisms may explain the sequential nature of rapid development seen in this case.

The chronology of cataracts presenting prior to the diagnosis of insulin-dependent diabetes along with the *WFS1* variant noted in our patient is unique but aligns with prior studies that identify *WFS1* gene dysfunction as a pathway for cataract formation converging with those related to diabetes mellitus. This case suggests that rapidly acquired cataract formation can result from dominant *WFS1*-SD and should be worked up with genetic testing in certain clinical contexts.

## Data Availability

The original contributions presented in the study are included in the article/supplementary material. Further inquiries can be directed to the corresponding author.
